# A Two-Layers Based Approach of an Enhanced-Map for Urban Positioning Support

**DOI:** 10.3390/s121114508

**Published:** 2012-10-29

**Authors:** Carolina Piñana-Díaz, Rafael Toledo-Moreo, F. Javier Toledo-Moreo, Antonio Skarmeta

**Affiliations:** 1 Department of Information and Communication Engineering, Facultad de Informática, Universidad de Murcia, Campus de Espinardo, Murcia, Spain; E-Mails: carolina.pinana@um.es (C.P.-D.); skarmeta@um.es (A.S.); 2 Department of Electronics and Computer Technology, Universidad Politècnica de Cartagena, ETSIT, Cartagena, Spain; E-Mail: javier.toledo@upct.es

**Keywords:** mapping, enhanced maps, positioning

## Abstract

This paper presents a two-layer based enhanced map that can support navigation in urban environments. One layer is dedicated to describe the drivable road with a special focus on the accurate description of its bounds. This feature can support positioning and advanced map-matching when compared with standard polyline-based maps. The other layer depicts building heights and locations, thus enabling the detection of non-line-of-sight signals coming from GPS satellites not in direct view. Both the concept and the methodology for creating these enhanced maps are shown in the paper.

## 1. Introduction

Many applications based on vehicle localization, such as navigation systems, fleet management or Electronic Toll Collection (ETC), are a reality today thanks to the so-called Global Navigation Satellite Systems (GNSS) and digital maps. GNSS devices are exploited to estimate the vehicle location, while digital maps are used to refer this location to the road segments where the vehicle drives.

However, location-based applications must face serious drawbacks in urban environments, where perhaps safety systems and location-based services become of more necessity. Main drawbacks can be summarized as follows:

- In urban built-up areas, the satellite signals used by GNSS sensors to estimate the vehicle location are strongly affected by the environment. GNSS signals are reflected, dispersed and attenuated by buildings, other vehicles, trees, *etc.* [1].
- While in highways and interurban areas the road layout trends to be simple and the most common approach to define the road shape based on polylines works well [2], in cities the road layout is far more complex and the polylines lack the necessary flexibility to accurately define the road shape. Therefore, standard digital maps are not always suitable as location reference.
- Map-matching algorithms must perform an extremely challenging task if we consider the large number of possible road segments to match the position of the vehicle, the GNSS errors and the lack of completeness and accuracy of the digital maps [3].

The concept of Enhanced Digital maps (EDmaps), also known as Enhanced Maps (Emaps), appeared with the purpose of creating better maps that could satisfy the needs of some vehicular applications with requirements of terms of map accuracy and completeness higher than those offered by standard maps [4]. Emaps are meant to be more complete and accurate than standard maps. To do so, Emaps may store more detailed data or some parameters that are not usual in standard maps based on polylines.

Our Emap proposal aims at supporting positioning and map-matching in urban areas, for which it stores information in two different layers:

- A road layer, dedicated to describe urban road layouts that is flexible enough to model complicated shapes. When developing Emaps, most of the authors focus their efforts on the accuracy of the centerline of the lane and the estimate of the road curvature. However, in this work another relevant aspect of the map is covered: the accurate representation (in our case, at submeter accuracy) of the road borders in an urban environment, which is contrary to the most common approach of depicting the centerlines, the number of lanes and their widths. This allows further possibilities in map-matching algorithms that can benefit from a more complete description of the road, providing more precise allocations of the vehicles, that are not necessarily referred to the centerline.
- An elevation layer that contains locations and heights of the buildings along the road. This way, when a vehicle is on a given point of the road, it will be feasible to create a visibility map of the GNSS satellites, detecting whether a satellite is in Line-Of-Sight (LOS) or in Non-Line-Of-Sight (NLOS). When solving the calculation of the vehicle positioning, NLOS satellites can be then removed, avoiding the biases introduced by the multipath effects caused by faulty measurements coming from NLOS satellites. Due to the elevation information stored in the map, the model presented in this paper is named Elevation-Enhanced map, or simply EEmap.

The rest of the paper goes as follows: Section 2 presents most relevant works published in this field. Section 3 introduces the EEmap concept and model. Next, Section 4 explains the creation process of the EEmap. Section 5 shows some relevant considerations in terms of accuracy and memory use. Finally, Section 6 concludes the paper.

## 2. Related Works

Due to its benefits, the concept of enhanced road map has been exploited in former works of the authors in order to achieve lane-level positioning [5], lane-change detection [6] or position integrity [7]. This work follows this research line, adding new contributions to this field.

Some other authors have exploited the information stored in navigable maps for map-aided positioning [8,9]. Also, new developments are under progress in the R&D teams of most map providers [10,11] to bridge the gap between the mapping standards and the requirements of advanced driving assistance applications (ADAS).

To the best of the authors' knowledge, there are no works in the literature that cover within the same EEmap concept the two problems addressed in this paper: modelling the vehicle drivable space, and supporting NLOS with a previously created digital map.

Most commonly, Emaps with an emphasis on the road layer focus on the accurate description of the road centerline or its curvature. Bétaille *et al.* [12] present a lane-level Emap based on clothoids with submeter accuracy at the lane-centerline, although complex crossroads and roundabouts are not included as a part of the Emap. Wang *et al.* in [13] include intersection points aiming at better describing the road curvature at them. Following a different approach, the French Institut National de l'Information Géographique et Forestière (IGN) has developed a triangle-based road/street model available only in certain areas, introducing an interesting alternative to the centerline based description [14]. Although triangles represent the most general geometrical shape for describing a surface, it is the authors' opinion that some other figures may represent the road shape with better accuracy and less memory demand.

With regard to the elevation layer, some related works such as [15] introduced the idea of creating a map of the obstacles for sky visibility, in this occasion applied to the railways domain. In [16], a fish-eye infrared camera was used to map the satellite positions with respect to the surrounding buildings. More recently, Marais *et al.* [17] addressed this issue for guided transport in urban environments, using also a fish-eye camera on the vehicle and aiming at building in real-time a 3D model from the successive images.

Contrary to the approach of creating visibility maps of the sky on the spot while driving, our work focuses on previously created and stored maps that can be accessed when needed. In particular, only the road elevation itself plus buildings are considered, since buildings are the elements that play the most relevant role in multipath effects [18–20]. In this line, Costa [18] employs a simulation model that includes a digital elevation model, building databases and a vegetation model to process an azimuth-elevation map of path states (clear, shadowed and blocked) for a large number of observers. This simulation model is exploited for studying and planning satellite-based location and navigation applications. In [20], the authors employ a sophisticated 3D city model to determine the building boundaries for GPS visibility, although the 3D model itself and its creation are not explained in the paper. Peyret *et al.* in [21] exploit a GIS map and a graphic tool to estimate the heights of the buildings, what is further applied to NLOS detection. The map employed in the tests carried out in [21] is a project of the French IGN that adds information about the buildings along the urban streets. At the time of developing our work, a few districts of France were covered by the IGN Batimap project. A comparison between this map and our proposal is presented later on in the paper.

Finally, let us remarks that a first concept of the elevation layer presented in this work was introduced in [22].

## 3. EEmap Concept

**Figure 1.**
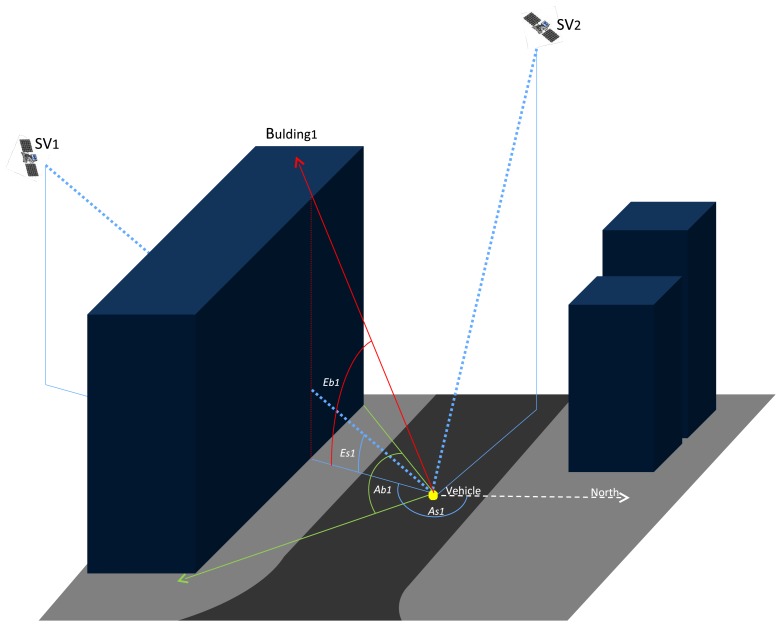
EEmap concept with relevant angles for the NLOS detection algorithm.

An schematic vision of the EEmap and its two layers is shown in Figure 1. The road layer is presented in dark grey and it depicts the road flooring, opposite to the light grey color of the sidewalks (or more precisely any non-drivable space). The characteristics of our road layer model makes it feasible to describe the surface of such shapes as the one presented in this figure, with both straight and rounded elements. This will be described in more details in Section 3.1. Buildings are drawn in blue and represent the obstacles that block the GPS satellite signals from reaching the receiver antenna in a direct ray. The principle of how this layer can support positioning is also shown in Figure 1. For a given vehicle position, the azimuth and elevation angles of each *i* GPS space vehicle (SV_i_) are calculated, respectively *As_i_*, *Es_i_*. Then, a first comparison between the *As_i_* and the blockage azimuth angle of a given *j* building, *Ab_j_*, is performed. If *As_i_* is within the *Ab_j_* interval, a second comparison is made between the elevation angle of *Es_i_* and the elevation angle of the building *Eb_j_*. In the image of Figure 1, examples of two different cases are represented. SV_1_ is blocked by building 1 and therefore SV_1_ is classified as in NLOS at that instant. On the contrary, no buildings block the SV_2_ signal and consequently this satellite is assumed to be in LOS. This is the concept of the NLOS detection algorithm based on our EEmap.

**Figure 2.**
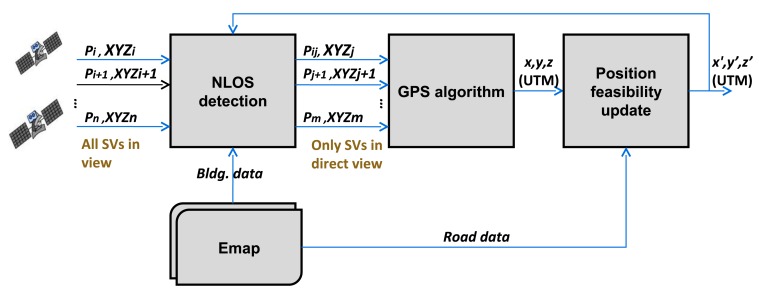
Positioning loop with the NLOS algorithm, the GPS algorithm and the position feasibility algorithm.

Both layers complement each other and may be exploited at different instants of the positioning and map-matching loop. A scheme of this process is presented in Figure 2. After the GNSS receiver collects measurements from tracked satellites, the NLOS detection algorithm decides whether a satellite is in LOS or in NLOS. Inputs of this algorithm are the satellite measurements, the EEMap, and a prior estimate of the vehicle position obtained for instance from a particle filter (PF). NLOS satellites are discarded, and only satellites in LOS are used as inputs of the second step, the GPS solving algorithm, that provides the position of the antenna. For this step, a Least Squares algorithm was developed and presented in [22]. After that, it is evaluated whether or not the estimated position belongs to the drivable road. Following the example of the PF, particles that are out of the road limits will be eliminated. This will lead to a new PF centroid that considers only feasible options. Finally, this best estimate is reintroduced in the positioning loop to be used in the next step. This way, the road layer of the EEmap supports the NLOS detection by adding more information into the loop, both horizontally (the vehicle shall be within the road bounds) and vertically (the vehicle shall be on the road and not over or under it). Consequently, thanks to the road layer, NLOS detection can be more accurate, resulting in a more accurate location estimate of the vehicle.

Although both EEmap layers can be exploited by independent algorithms with different purposes, it is the authors' intent to present them as a single EEmap concept, in which the interaction between both layers may bring extended benefits to the positioning estimate thanks to the redundant information.

In the next two sections, the principles of both the road and the elevation layers models are described.

### 3.1. Road Layer

The concept of road layer is based on a small set of plane figures located in a 3D environment and defined by a certain number of descriptors. Each figure represents a portion of roadway and the succession of these figures arranged next to one another defines the complete form of the road.

**Figure 3.**
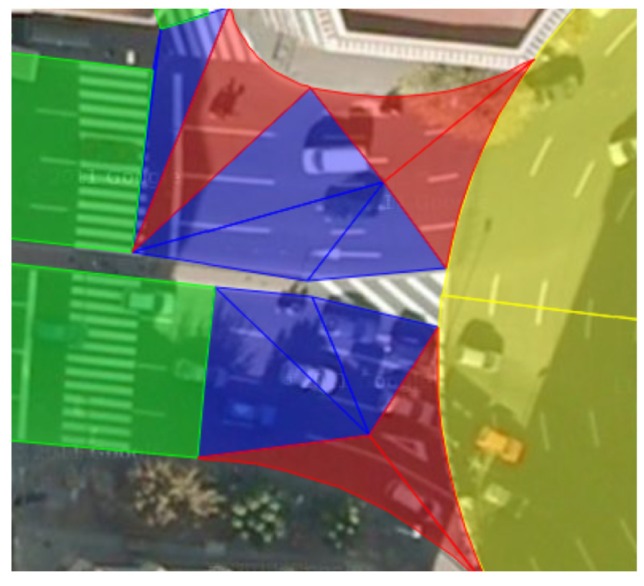
Superposition of some EEmap figures (in green, blue, red and yellow) over the roads of the city of Murcia, Spain.

In our model, there are four basic types of figures to describe the road shape. Figure 3 shows a small area of the city of Murcia, Spain, where these four elements are depicted. The next sections describe these figures in detail.

#### 3.1.1. Trapeze

**Figure 4.**
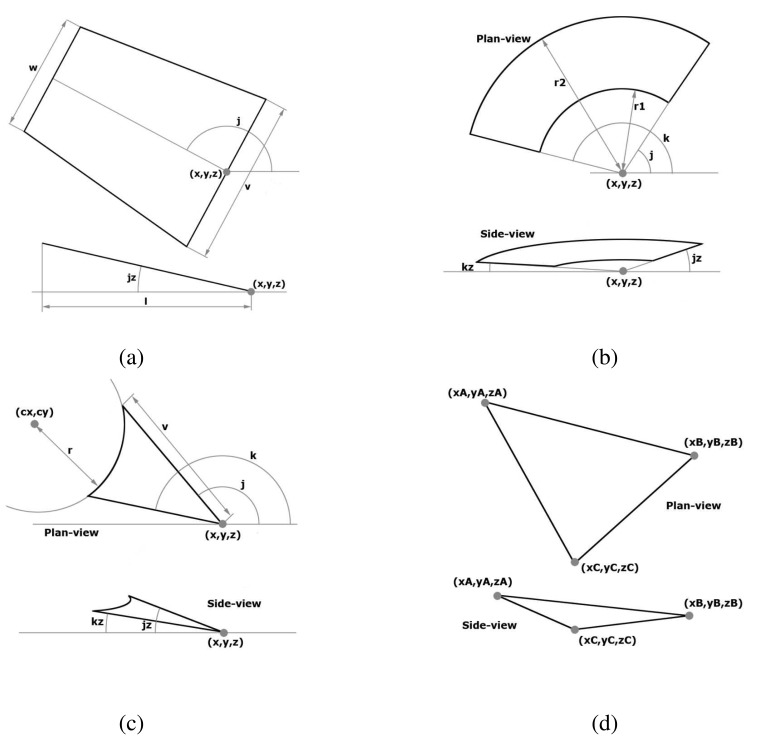
EEmap road figures with descriptors.

This figure is used to define straight portions of the road (see green figures in Figure 3). These stretches can vary in width along the longitudinal axis, which is being modelled by adapting the width of the trapeze bases *v* and *w*, as shown in Figure 4(a). Its descriptors are as follows:

- *w*: width of the base opposite to the reference point.
- *v*: width of the base containing the reference point.
- *x, y, z*: coordinates East, North, Elevation (UTM) of the central point of the base.
- *j*: argument (between 0 and 2*π*) of the vector joining the midpoints of the bases with respect to the plane *XY*.
- *jz*: argument (between +*π*/2 and −*π*/2) of the vector joining the midpoints of the bases with respect to the *Z* axis.
- *l*: distance of the projection of the trapeze height with respect to the plane *XY*.

#### 3.1.2. Crown Sector

The crown sector aims to describe roundabouts with both an outer and an inner radius that adjust to the width of the road (see yellow figures in Figures 3 and 4(b) for a description of its parameters). Values of *j* and *k*, measured in radians, are used to determine the argument of the polar coordinates of the crown lateral bounds in the radial direction of the crown sector with respect to its center. The descriptors of a crown sector are:

- *x*, *y*, *z*: coordinates East, North, Elevation (UTM) of the central point of the roundabout.
- *j*: argument (between 0 and 2*π*) of the vector joining one side of the crown to the center of the roundabout.
- *k*: argument (between 0 and 2*π*) of the vector from the other side of the crown to the center of the roundabout.
- *jz*: argument (between +*π*/2 and −*π*/2) of the vector from the center of the roundabout to the side described by *j* with respect to the *Z* axis.
- *kz*: argument (between +*π*/2 and −*π*/2) of the vector from the center of the roundabout to the side described by *k* with respect to the *Z* axis.
- *r*1: radius of the inner arc.
- *r*2: radius of the outer arc.

#### 3.1.3. Arrowhead

The borders of the road in an intersection are rarely composed of sharp-pointed or well-defined angles but are described by rounded shapes (see red figures in Figure 3). This figure is similar to a triangle, but having one of its sides curved, making it easy to describe those portions of the road by varying point *(cx, cy)* and radius *r* as shown in Figure 4(c).

- *x, y, z*: coordinates East, North, Elevation (UTM) of the main vertex (between both straight sides).
- *j*: argument (between 0 and 2*π*) of the vector that describes one side of the arrowhead with respect to the main vertex in the plane *XY*.
- *k*: argument (between 0 and 2*π*) of the vector that describes the other side of the arrowhead with respect to the main vertex in the plane *XY*.
- *jz*: argument (between +*π*/2 and −*π*/2) of the vector described by *j* with respect to the *Z* axis.
- *kz*: argument (between +*π*/2 and −*π*/2) of the vector described by *k* with respect to the *Z* axis.
- *cx*, *cy*: coordinates East, North (UTM) of the center of the circle that describes the lateral curve.
- *r*: radius of the arc whose center is *cx, cy*.
- *v*: this parameter represents the largest of the lengths of the straight sides in the arrowhead. Although its inclusion is not necessary, it is introduced to improve the computational efficiency when checking if a point is inside the figure.

#### 3.1.4. Triangle

Since the triangle is the cornerstone of any polynomial geometric figure, its inclusion is necessary to complete the remaining portions of the roadway that cannot be well depicted by the three aforementioned EEmap elements (see blue triangles in Figure 3). The triangle data descriptors define its vertices in a three-dimensional environment (Figure 4(d)).

- *xA, yA, zA*: coordinates East, North, Elevation (UTM) of the vertex *A*.
- *xB, yB, zB*: coordinates East, North, Elevation (UTM) of the vertex *B*.
- *xC, yC, zC*: coordinates East, North, Elevation (UTM) of the vertex *C*.

### 3.2. Elevation Layer

The elevation layer stores information of the buildings' location and heights. For each building, the UTM coordinates of the two corners nearest to the road are stored, as well as its width and height (Figure 1). The description of the buildings follows the format given in Table 1, where subscripts 1 and 2 stand for the 2D position ends of the facade under consideration. In this approach, both corners of the facade share a single value of altitude. This can be done since buildings are normally horizontal, even if the road is tilted, and it will be the roof of the building (and not the ground) that defines the sky visibility.

## 4. EEmap Creation

### 4.1. Road Layer

**Figure 5.**
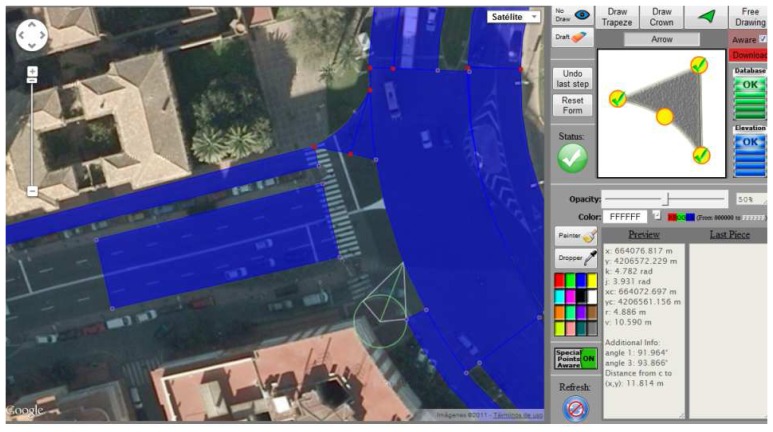
Overview of the web tool developed to create the road layer of the EEmap. On the right, the toolbar and drawing assistant; on the left, the map drawing overlaying the aerial image of an urban area of Murcia, Spain.

The creation of this layer is carried out by means of computer-assisted photogrammetry. To do so, a specific web tool that was presented in [23] was created. The application uses AJAX technology [24] to maintain communication with the server, and contains a database where the descriptors of the figures of the map are stored. The images used to carry out the task of photogrammetry are supplied by Google Maps, offering a resolution in the satellite images suitable for the purpose of this work. This web tool is quite simple and fast to use without detriment to the accuracy. The process of map-making exploits a set of drawing tools of the web application that assist the manual draw of figures on a map image. An overview of it is shown in Figure 5.

### 4.2. Elevation Layer

Two methods for extracting the model parameters of the buildings presented in Table 1 were developed and are presented in the next sections. The first method, named Building Image-based Method, depends on the complete visibility of the building. Building images are obtained from Google Earth using the Street Viewer tool. This allows fast prototyping, avoiding extensive field campaigns for extracting the building features. However, since the complete view of the building is not always available, a second method named Story-based Method based on the number of stories is proposed. It consists in applying the same art as the “Building Image-based Method” in images that only show isolated parts of buildings, such as the ground or the first floor, something common in urban canyons. In subsequent discussions, a comparison of both methods will show the consistency of our approach.

#### 4.2.1. Building Image-Based Method

A dedicated algorithm based on Google Earth images processing is proposed for simply and efficiently obtaining building heights. The first step employs a frontal view of the building facade provided by the Street Viewer tool of Google Earth. Then, a border detector algorithm is implemented to get an edge intensity image. The small-scale model obtained with the low-level detector provides edge information of the scene, which is used to calculate the relationship between the width and the height of the building. Since its real width can be measured in the aerial image provided by Google Earth, it is possible to extrapolate feature information to calculate the real height of the building. As it will be shown later on in the paper, experimental results show that the proposed algorithm works well in cases where a complete frontal view of the building is available in the Street Viewer tool of Google Earth.

#### 4.2.2. Story-Based Method

An entire view of the facade of the building is not always available in Google Earth, especially in narrow streets with limited visibility where the only visible features correspond to the ground and first floors. In these cases, it is possible to use the prior algorithm to detect edges only of available parts and then extrapolate measurements to the whole building just by counting the number of stories. Given that the heights of the regular stories of a building are all the same, by applying this technique the height of the entire building can be computed as follows:

$$h=k_{1}\times s+k_{2}$$

where *s* denotes the number of stories, *k*_1_ represents a constant value of the height of an arbitrary floor and *k*_2_ is the height of the ground floor along with any extra element not included in a standard story of the building. Both parameters are obtained with the detector estimation method.

## 5. Considerations of Accuracy and Memory Use

The EEmap concept presented in this paper will be valid as long as the information stored in it is accurate enough and the corresponding memory needs will not be a stopper. These two aspects are analyzed in the next sections.

### 5.1. Accuracy

#### 5.1.1. Road Bounds

The main purpose of this layer is to describe urban roadways with better accuracy than standard maps based on polylines. In particular, since the area to be mapped is modelled with figures that adapt to the road shape, the borders of the roadways are defined with much more accuracy. This results in interest for vehicle positioning and map-matching algorithms, especially when techniques based on particle filters, bounded-errors, interval analysis or belief theory are used [7,25].

The final accuracy of the EEmap depends on several factors:

- The quality and accuracy of the aerial image. The web tool developed for EEmap creation is supported by Google Maps. In the tests made in several cities of Spain, its quality was found sufficient to achieve at least sub-meter accuracy at the road border. Although there may be some absolute errors caused by drifts in the aerial view, in the tests carried out no significant errors were found. Nevertheless, solving this problem is out of the scope of this work. In addition, occasionally trees and other objects may block the view of the road border although these situations are very unusual.
- Model limitations. The figures presented in this work have been selected because they can represent the road shape with good accuracy and they complement one another. In addition to that, the computational complexity of determining whether certain UTM coordinates are within the figures was also taken into consideration. Due to the limited number of figures, there may be situations where the suitability is not optimal. Although these situations are unusual in urban environments, even in these cases the intended accuracy can be achieved by means of modelling the road with the option *free drawing*, where the polygon entered by the user is automatically broken down in an optimal number of triangles.
- The operator skills. As with any other human-driven process, the capabilities of the EEmap web tool user to develop the best strategies will condition the final quality of the EEmap. Nevertheless, it is the authors' belief that the tool developed is simple to use.

The benefits in terms of accuracy achieved when characterizing urban roadways with the EEmap model compared with the conventional maps based on polylines depend on the area under consideration, the shape of its roadway and the quality of the standard maps there.

**Figure 6.**
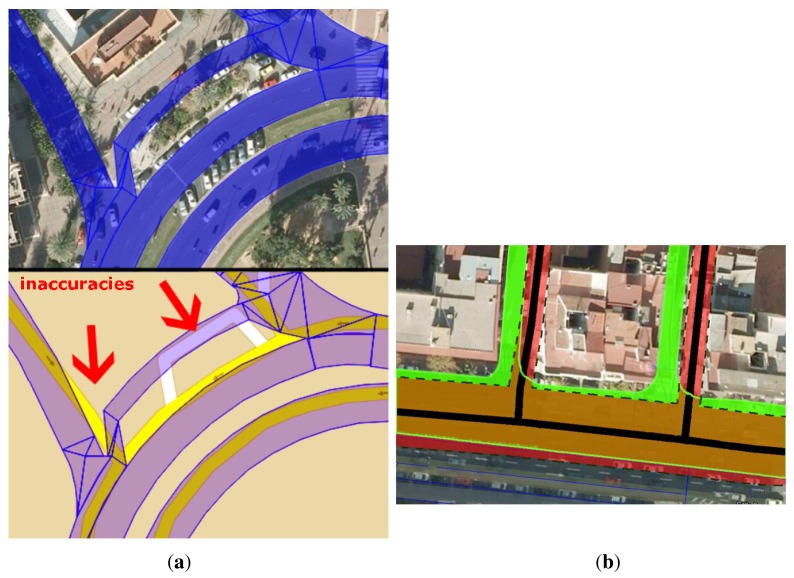
(**a**) Top: aerial view of the scenario under consideration with superimposed EEmap (in blue); Bottom: inaccuracies in the polyline-based map with an overlaying EEmap (in blue). (**b**) Black: centerline of the polyline-based map. The inaccuracies committed in the assumption of a fixed lane-width can be seen (superimposed in red) together with the imprecision of the mid-lane. Black-dash lines show the limit of these lanes. Green: superimposed EEmap.

Figure 6(a) shows an example of an interesting area near a roundabout. In the upper image, the EEmap (in blue) is superimposed on the aerial view of the area. In the lower image, the polyline-based map is displayed together with the EEmap figures in blue. It can be observed that the polyline-based map differs from the middle of the street, which would add errors in map-matching. Some errors caused by the map model are also visible (remarked with red arrows in the image).

Figure 6(b) shows another example of this situation. At the bottom of the image, lanes on the direction from right to left are represented in the commercial map with a common centerline (solid black) and the road width (black dashed line). Two intersecting roads are also depicted in the same way. The EEmap of this area is superimposed in green. Brown areas are overlaps between both the commercial and the enhanced maps.

In our tests performed in the South of Spain, it was usual to detect errors of several meters in commercial city maps. Most commonly, errors are at the level of 4 or 5 meters, but in some situations its value can reach more than 10 meters. These error values in the maps entail insuperable barriers for positioning and map-matching algorithms, especially when lane-level is aimed.

#### 5.1.2. Elevation

As aforementioned, two different methods named building image-based method and story-based method are used to create the EEmap, depending on the visibility of the buildings in the Google street viewer. The former will be used when there is good visibility of a building in the Google Street Viewer, and the latter will be used when only the height of the lower stories and its number are available. To disentangle the possible influence of using one method or another on the accuracy of the EEMap, both techniques have been compared with buildings located in Murcia, Spain. A sample of the results is given in Table 2. As it can be seen, the difference between heights computed using one or another method is only slightly different with a median relative difference of 0.0048. Therefore, it can be assessed that the use of one method or another makes no significant difference.

The goodness of the buildings' measurements obtained following our methodology has been validated by using the data of the French IGN Batimap, formerly introduced in Section 2. The differences in ten buildings of France are shown in Table 3. Heights are always consistent, and the relative difference between both datasets is always lower than 0.05 with an average of 0.032. In absolute terms, maximum differences are around half a meter.

### 5.2. Memory Use

#### 5.2.1. Road Layer

**Figure 7.**
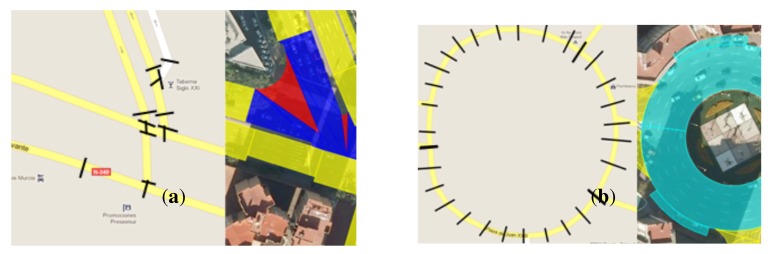
(**a**) Situation where the use of our EEmap leads to an increase in memory requirements. Left image: polyline-based map where black dashes indicate nodes or shape points; Right image: EEmap of the same area. (**b**) Example of a roundabout area where the figure-based EEmap uses a lower number of figures than a polyline-based map. Left image: polyline image with black dashes that separate the lines at the shape points; Right image: EEmap split of the same area that uses less parameters.

With regard to the memory storage of maps, enhanced maps usually require more memory than conventional cartographic systems. This is because a larger amount of data descriptors is needed. An example of this can be seen in Figure 7(a). This example has been used to perform a numerical comparison between a polyline-based map and our EEmap. To do so, it is assumed that polylines employ four descriptors (*x, y, z* and lane width) to describe the stretches between nodes and/or shape points (separated by black dashes in the image of Figure 7(a)), with the exception of the last section that will also have three extra values, *x, y, z*, corresponding to the end of the polyline. Table 4 shows the results of this comparison. While 264 values are needed to describe this area with the EEmap, the polyline-based map requires only 78 values. However, depending on the shape of the road, the use of our EEmaps can lead to a reduction of the amount of memory use, as shown in Table 5. This table shows the results obtained when carrying out the same analysis made in Table 4, but this time for the image presented in Figure 7(b). In this case, only 36 values are needed to describe the roundabout with the new enhanced maps, while 123 values are needed in order to make a description based on polylines.

A detailed comparison of the demanded storage capacity is strongly dependent on the layout of the urban roadways. Nevertheless, since in most cases the description based on an EEmap will cause higher memory occupation, the decision of whether its exploitation is worthy will depend on the accuracy requirements of the intended application.

#### 5.2.2. Elevation Layer

The memory needed to store the elevation layer of an EEmap depends on the density of buildings in the area under consideration. Since the focus of this paper is urban scenarios, let us consider the case of a dense built-up area.

Along one kilometer of street in the city center of Murcia, Spain, we counted 139 relevant facades considering both sides of the road. For this count, relevant facades are those that could block the direct view of a GPS satellite from a receiver installed in a vehicle on this road. Typically, for a building with four facades, only three are relevant since the fourth is hidden behind the other three. Each facade needs eight parameters according to Table 1, which results in 1112 parameters per kilometer in a highly dense built-up scenario.

Considerations about the compression of these data or its optimization are out of the scope of this paper.

## 6. Conclusions

A new model of enhanced map, called Elevation-Enhanced map (EEmap), was presented. Our EEmap is based on two layers. The first layer, which is the road layer, includes accurate information of the road bounds, thus enabling advanced positioning and map-matching techniques to provide more accuracy and consistency. Secondly, the elevation layer complements the road layer with the description of the elevation environment, which in urban areas (the focus on this paper) consists of mostly buildings. By knowing the location and height of the buildings, an azimuth-elevation map of the GPS satellites visibility can be built for a given vehicle position. Therefore, satellites in direct view (LOS) can be distinguished from those in NLOS, supporting multipath detection and elimination. The concept and the creation process of the EEmap have been presented in the paper. The paper is completed with the analysis of accuracy and memory use for both layers of the enhanced map. Results show how the concept and methodology for creating EEmaps can provide accurate maps of urban roads and its environment that are also affordable in terms of memory use.

## Figures and Tables

**Table 1.** Building model parameters.

*Bldg Id*	*Up*	*East*_1_	*North*_1_	*East*_2_	*North*_2_	*w*	*h*

**Table 2.** Comparison of building height estimates between image-based method and story-based method.

**Bldg Id**	**Method 1**	**Method 2**	**Absolute Difference**	**Relative Difference**
1	26.27	26.36	0.09	0.0034
2	38.99	38.70	0.29	0.0074
3	26.65	26.85	0.20	0.0075
4	25.07	25.01	0.06	0.0025
5	19.92	19.98	0.06	0.0033

**Table 3.** Comparison of building height estimates with the French IGN Batimap reference.

**Bldg Id**	**Method 1**	**Method 2**	**Absolute Difference**	**Relative Difference**
1	13.40	13.98	0.58	0.043
2	15.32	15.89	0.57	0.037
3	15.20	15.16	0.33	0.002
4	15.60	15.71	0.11	0.007
5	14.29	14.62	0.33	0.023
6	5.79	6.06	0.27	0.047
7	15.71	15.42	0.29	0.018
8	14.99	15.39	0.40	0.027
9	7.60	7.06	0.54	0.071
10	14.66	15.28	0.62	0.042

**Table 4.** Comparison of the number of parameters needed to describe the image shown in Figure 7(a) with a polyline-based map and our EEmap.

**Map Type**	**Elements**	**Descriptors by element**	**Total Descriptor**
Polyline-based	29 segments	4	
Map	6 final segments	7	78
Figure-based	10 Trapezes	8	
EEmap	18 Triangles	9	264
	2 Arrowhead	11	

**Table 5.** Comparison of the number of parameters needed to describe the image shown in Figure 7(b) with a polyline-based map and our EEmap.

**Map Type**	**Elements**	**Descriptors by element**	**Total Descriptor**
Polyline-based	29 segments	4	
Map	1 final segment	7	123
Figure-based	4 Crown	9	36
EEmap	Sectors		

## References

[1] Le Marchand O., Bonnifait P., Iba nez-Guzmán J., Bétaille D., Peyret F. (2009). Characterization of GPS multipath for passenger vehicles across urban environments. ATTI dell'Istituto Italiano di Navigazione.

[2] Quddus M.A., Ochieng W.Y., Noland R.B. (2007). Current map-matching algorithms for transport applications: State-of-the-art and future research directions. Elsevier Trans. Res. Part C.

[3] Grush B. (2008). The case against map-matching. Eur. J. Navig..

[4] Janausch T., Gern A., Linder F., Maile M., Wilson C., Wolermann B., Pilutti T., Ahmed-Zarid F., Palmer M., Shulman M., Waldis A., Sadekar V., Deering R., Grimm D., Hamilton B., Kellum C., Krishnan H., Haskitt P., Nyczak G., Uehara Y., Goudy R., Sanislow D., Torres R. (2004). Enhanced Digital Mapping Project—Final Report.

[5] Toledo-Moreo R., Bétaille D., Peyret F., Laneurit J. (2009). Fusing GNSS, dead-reckoning and enhanced maps for road vehicle lane-level navigation. IEEE J. Sel. Top. Signal Process..

[6] Toledo-Moreo R., Zamora-Izquierdo M.A. (2009). IMM-based lane-change prediction in highways with low-cost GPS/INS. IEEE Trans. Intell. Transp. Syst..

[7] Toledo-Moreo R., Bétaille D., Peyret F. (2010). Lane-level integrity provision for navigation and map matching with GNSS, dead reckoning, and enhanced maps. IEEE Trans. Intell. Transp. Syst..

[8] Cui Y., Ge S.S. (2003). Autonomous vehicle positioning with GPS in urban canyon environments. IEEE Trans. Robot. Autom..

[9] Fouque C., Bonnifait P., Bétaille D. Enhancement of Global Vehicle Localization Using Navigable Road Maps and Dead-Reckoning.

[10] Zott C., Yuen S.Y., Brown C.L., Bertels C., Papp Z., Netten B. Safespot Local Dynamic Maps: Context-Dependent View Generation of a Platform's State and Environment.

[11] Wevers K., Dreher S. Digital Maps for Lane Level Positioning.

[12] Bétaille D., Toledo-Moreo R. (2010). Creating enhanced maps for lane-level vehicle navigation. IEEE Trans. Intell. Transp. Syst..

[13] Wang C., Hu Z., Uchimura K. A Precise Road Network Modeling and Map Matching for Vehicle Navigation.

[14] Institute National de L'Information Geographique et Forestier http://www.ign.fr/.

[15] Marais J., Berbineau M., Frimat O., Franckart J.P. A New Satellite-Based Fail-Safe Train Control and Command for Low Density Railway Lines.

[16] Meguro J.I., Murata T., Takiguchi J.I., Amano Y., Hashizume T. (2009). GPS multipath mitigation for urban area using omnidirectional infrared camera. IEEE Trans. Intell. Transp. Syst..

[17] Marais J., Ambellouis S., Flancquart A., Lefebvre S., Meurie C., Ruichek Y. (2012). Accurate localisation based on GNSS and propagation knowledge for safe applications in guided transport. Transp. Res. Arena.

[18] Costa E. (2011). Simulation of the effects of different urban environments on GPS performance using digital elevation models and building databases. IEEE Trans. Intell. Transp. Syst..

[19] Marais J., Berbineau M., Heddebaut M. (2005). Land mobile GNSS availability and multipath evaluation tool. IEEE Trans. Vehicular Technol..

[20] Groves P., Wang L., Ziebart M. Shadow matching: Improved GNSS accuracy in urban canyons. GPS World.

[21] Peyret F., Bétaille D., Florian M. Non-Line-of-Sight GNSS Signal Detection Using an on-Board 3D Model of Buildings.

[22] Pinana-Diaz C., Toledo-Moreo R., Bétaille D., Gomez-Skarmeta A. GPS Multipath Detection and Exclusion with Elevation- Enhanced Maps.

[23] López-Pérez D., Toledo-Moreo R. An Approach to Road Enhanced Maps in Urban Areas.

[24] Garrett J.J. Ajax: A New Approach to Web Applications. http://AdaptivePath.com.

[25] Abdallah F., Nassreddine G., Denoeux T. (2012). A multiple-hypothesis map-matching method suitable for weighted and box-shaped state estimation for localization. IEEE Trans. Intell. Transp. Syst..

